# Activation of Human γδ T Cells: Modulation by Toll-Like Receptor 8 Ligands and Role of Monocytes

**DOI:** 10.3390/cells9030713

**Published:** 2020-03-13

**Authors:** Ruben Serrano, Daniela Wesch, Dieter Kabelitz

**Affiliations:** Institute of Immunology, University Hospital Schleswig-Holstein Campus Kiel, D-24105 Kiel, Germany; ruben.serrano@uksh.de (R.S.); daniela.wesch@uksh.de (D.W.)

**Keywords:** gamma delta T cells, inflammasome, monocytes, phosphoantigen, Resiquimod, Toll-like receptors, zoledronic acid

## Abstract

Background: Human Vγ9Vδ2 γδ T cells can kill a variety of cancer cells and have attracted substantial interest for cancer immunotherapy. Toll-like receptor (TLR) ligands are promising adjuvants for cancer immunotherapy, but TLR7/8 ligand Resiquimod has been shown to inhibit CD4 T-cell activation in a monocyte-dependent manner. Therefore, we studied the modulation of human γδ T-cell activation by TLR7/8 ligands. Methods: Peripheral blood mononuclear cells (PBMC) or purified γδ T cells together with purified monocytes were stimulated with zoledronic acid or phosphoantigens in the absence or presence of various imidazoquinoline TLR7 or TLR8 agonists. Read-out systems included interferon-γ induction and cellular expansion of γδ T cells, as well as viability, cell surface antigen modulation, and IL-1β and TNF-α production of monocytes. Results: TLR8 ligand TL8-506 and TLR7/8 ligand Resiquimod (but not TLR7 ligands) rapidly induced IFN-γ expression in γδ T cells within PBMC, and co-stimulated phosphoantigen-induced IFN-γ expression in γδ T cells. On the other hand, TLR8 ligands potently suppressed γδ T-cell expansion in response to zoledronic acid and phosphoantigen. Purified monocytes secreted large amounts of IL-1β and TNF-α when stimulated with TLR8 ligands but simultaneously underwent substantial cell death after 24 h. Conclusions: TLR8 ligand-activated monocytes potently co-stimulate early γδ T-cell activation but failed to provide accessory cell function for in vitro expansion of γδ T cells.

## 1. Introduction

The major subset of γδ T cells in human peripheral blood expresses the T-cell receptor (TCR) variable elements Vγ9 paired with Vδ2 (Vγ9Vδ2; hereafter referred to as Vγ9 or Vδ2). Vδ2 T cells recognize in a TCR-dependent manner pyrophosphate intermediates of the eukaryotic and prokaryotic pathways of cholesterol synthesis, collectively termed phosphoantigens (pAg). In eukaryotic cells, intermediates of the mevalonate pathway, notably isopentenyl pyrophosphate (IPP), have been shown to selectively activate Vδ2 T cells, but high concentrations of IPP (in the micromolar range) are required [1,2]. While normal cells do not produce enough IPP to activate γδ T cells, many transformed and tumor cells have a dysregulated mevalonate pathway and produce increased amounts of IPP, thereby leading to Vδ2 γδ T-cell activation [3,4]. Homologous pyrophosphates are produced by prokaryotes including many bacteria and some parasites in the non-mevalonate (or Rohmer’s) pathway of cholesterol synthesis. The non-mevalonate intermediate (*E*)-4-Hydroxy-3-methyl-but-2-enyl pyrophosphate (HMBPP) activates human Vδ2 T cells at pico- to nanomolar concentrations [5]. Synthetic homologs of naturally occurring pAg like bromohydrin pyrophosphate (BrHPP) are also potent and selective activators of Vδ2 T cells [6]. The recognition of homologous microbial and tumor-derived pAg by the same Vγ9Vδ2 TCR forms the basis for the role of Vδ2 T cells in both anti-infective and anti-tumor immunity [7,8].

The recognition of pAg by Vδ2 T cells does not involve class I or class II human leukocyte antigens (HLA) but strictly depends on the presence of transmembrane butyrophilin (BTN) molecules. Specifically, it has been shown that BTN3A isoforms are indispensable for the activation of Vδ2 T cells [9]. While the precise molecular pathway is still under debate, the current model implies that pAg bind to the intracellular B30.2 signaling domain of BTN3A1, thereby (via recruitment of adaptor proteins) inducing a conformational change of the extracellular domain of BTN3A1 which is then somehow recognized by the Vγ9Vδ2 TCR [10]. Very recently, however, it was reported that other isoforms of BTN, including BTN2A1, are also involved in the pAg-dependent activation of Vδ2 T cells [11].

In addition to pAg, nitrogen-containing aminobisphosphonates like zoledronic acid (ZOL) are potent indirect activators of Vδ2 T cells. ZOL inhibits the farnesyl pyrophosphate synthase in the mevalonate pathway of cholesterol synthesis, leading to an upstream accumulation of IPP and subsequent Vδ2 T-cell activation and proliferation in the presence of exogenous IL-2 [2]. While neutrophils can also take up ZOL, only monocytes are potent producers of IPP among blood leukocytes, and are therefore essential accessory cells for Vδ2 T-cell activation by ZOL and related aminobisphosphonates [12]. Monocytes are also potent accessory cells for γδ T-cell activation by pAg like HMBPP and BrHPP. Presumably, exogenously added pAg also require binding to the intracellular B30.2 domain of BTN3A1 to activate Vδ2 T cells; however, the mechanism of cellular entry of pAg has not yet been identified.

Toll-like receptors (TLR) are multiligand receptors that recognize microbial cell wall-associated ligands and other pathogen-associated molecular patterns (PAMPs) but also damage-associated molecular patterns (DAMPs) like high-mobility-group-protein B1 (HMBG1) [13,14]. Monocytes express a variety of pattern recognition receptors, and different signaling pathways are triggered in response to the corresponding ligands. Among others, monocytes express TLR2,4,8,9 and produce pro-inflammatory cytokines like interleukin (IL)-1, IL-6, and tumor necrosis factor-α (TNF-α) [15,16]. Secretion of IL-1β requires activation of the inflammasome and the proteolytic cleavage of pro-IL-1β by caspase-1 to generate mature IL-1β [17]. In contrast, secretion of TNF-α proceeds via proteolytic cleavage of membrane-bound TNF-α and does not involve inflammasome activation [18]. Ligands for some TLRs like TLR2 (di- or triacetylated lipopeptides) and TLR4 (lipopolysaccharide, LPS) prime monocytes for IL-1β production (i.e., induce pro-IL-1β); however, IL-1β secretion requires additional inflammasome activation, e.g., by the bacterial toxin nigericin [17]. In contrast, IL-1β (and TNF-α) secretion can be stimulated by TLR8 ligands in human monocytes also in a caspase-1 independent pathway [19,20]. Importantly, inflammasome activation in monocytes triggers pyroptosis, a highly inflammatory form of programmed cell death [21]. Certain TLR7/8 agonists have been shown to promote anti-tumor immunity by enhancing the pro-inflammatory tumor micromilieu [22,23]. Moreover, several studies have demonstrated that TLR7/8 ligands modulate human T-cell activation in a monocyte-dependent manner. In the absence of monocytes, activation of purified T cells by anti-CD3 monoclonal antibodies (mAb) was co-stimulated by TLR7/8 ligand Resiquimod [24], whereas T cell activation was inhibited in the presence of Resiquimod-treated monocytes [24,25]. The inhibitory effect of Resiquimod-treated monocytes was reportedly not due to monocyte cell death but to the induction of an immune-suppressive monocyte phenotype involving adenosine, upregulation of programmed death-ligand (PD-L)1, and indoleamine 2,3-dioxygenase activity [25]. We previously reported that TLR3 ligand polyinosinic-polycytidylic acid (poly I:C) can directly co-stimulate human γδ T-cell activation [26]. Moreover, we also observed that TLR7 ligand imiquimod enhanced the susceptibility of tumor cell lines to killing by Vδ2 T cells [27]. However, the influence of TLR7/8 ligands on human γδ T-cell activation has not yet been investigated. In view of the potential application of Vδ2 T cells as effector cells in cell-based cancer immunotherapy [28,29], we investigated the modulation of the activation and proliferative Vδ2 expansion by TLR8 and TLR7/8 ligands.

## 2. Materials and Methods

### 2.1. Isolation of Peripheral Blood Mononuclear Cells and Purification Of Monocytes and γδ T Cells

Peripheral blood mononuclear cells (PBMC) were isolated from leukocyte concentrates of healthy adult blood donors obtained from the Institute of Transfusion Medicine, University Hospital Schleswig-Holstein (Kiel, Germany). Written informed consent was obtained from all donors. The research was conducted in accordance with the Declaration of Helsinki and was approved by the relevant institutional review boards (ethic committee of the Medical Faculty of the University of Kiel, Germany; code number: D546/16). PBMC were isolated by Ficoll-Hypaque density gradient centrifugation. γδ T cells were purified from PBMC by positive magnetic selection using the anti-TCRγ/δ micro-Bead Kit from Miltenyi Biotec (Bergisch Gladbach, Germany) following the instructions of the company. The procedure includes labeling of the γδ T cells with a specific hapten-coupled anti-TCRγδ mAb followed by staining the cells with FITC-labeled anti-hapten microbeads. To increase the purity of γδ T cells, two consecutive magnetic columns were applied. The purity of isolated γδ T cells was >97%. Monocytes were isolated from PBMC by negative isolation using the Pan Monocyte Isolation Kit following the instructions of the company (Miltenyi Biotech). Negatively isolated monocytes contained >96% CD14^+^ monocytes and <1% contaminating CD3^+^ T cells. The purity of isolated cells was assessed with flow cytometry.

### 2.2. Cell Cultures

PBMC (1.5 × 10^5^ per well) or purified γδ T cells (2 × 10^4^ per well) with or without purified monocytes (5 × 10^4^ per well) were cultured in wells of 96-well round bottom microtiter plates (Nunc; Thermo Fisher Scientific, Waldham, MA, USA) as detailed in the Results section. Culture medium was RPMI 1640 (Thermo Fisher Scientific) supplemented with antibiotics (100 U/mL penicillin, 100 µg/mL streptomycin) and 10% of heat-inactivated low endotoxin fetal bovine serum (Bio&Sell, Feucht, Germany). Stock solutions of the following TLR agonists (Invivogen, Toulouse, France) were prepared as recommended by the manufacturer, and were stored at −20 °C until use: Imiquimod (TLR7), CL264 (TLR7), ultrapure LPS (TLR4), TL8-506 (TLR8), Resiquimod (TLR7/8). The TLR8-specific RNA ligand ssRNA40 and the negative control ssRNA41 were also obtained from Invivogen. ssRNA41 also binds to TLR8 but does not induce signaling. The TLR8 agonist Motolimod (VTX-2337) was purchased from Sellekchem (Houston, TX, USA). Zoledronic acid (ZOL) and recombinant human IL-2 (Proleukin) were kindly provided by Novartis (Basel, Switzerland), (*E*)-4-Hydroxy-3-methyl-but-2-enyl pyrophosphate (HMBPP) was purchased from Echelon Biosciences (Salt Lake City, UT, USA). Bromohydrin pyrophosphate (BrHPP) [6] was kindly provided by Innate Pharma (Marseille, France). Final concentrations of ZOL, HMBPP, and BrHPP were 2.5 μM, 10 nM, and 300 nM, respectively [12]. TLR agonists were used at pre-determined concentrations as indicated under Results. Where indicated, cell cultures were supplemented with IL-2 (50 IU/mL). Short-term Vδ2 T-cell lines were established from PBMC as previously reported [30]. Briefly, PBMC were stimulated with 2.5 μM ZOL and 50 IU/mL IL-2. IL-2 was added every other day, and cell cultures were split every 2–3 days starting at day 6. Following this protocol, the purity of γδ T-cell lines after 14 days was routinely >95% CD3^+^Vδ2^+^. For intracellular analysis of interferon-γ (IFN-γ) expression, 4 × 10^5^ PBMC per well were cultured for 24 h in round-bottom microtiter plates in the presence or absence of TLR ligands and HMBPP (without exogenous IL-2). 33 μM monensin was added during the last 4 h to prevent cytokine secretion.

### 2.3. Determination of γδ T-Cell Expansion

Absolute numbers of viable Vγ9 T cells per microculture well were measured after 6 to 8 days by a flow cytometry-based method termed standard cell dilution assay (SCDA) [31]. In brief, cultured cells from 96-well round-bottom plates were washed and stained with anti-CD3-PE (clone SK7; BD Biosciences, Heidelberg, Germany) and anti-Vγ9-AF488 (clone 7A5 [32]) mAb. After one washing step, cells were resuspended in sample buffer containing a defined number of allophycocyanin (APC)-labeled fixed standard cells and 0.2μg/mL propidium iodide (PI). Standard cells were purified CD4 T cells that had been stained with APC-labeled anti-HLA class I mAb w6/32 and anti-TCRαβ mAb IP26, and thereafter had been fixed in 1% paraformaldehyde. Based on the known number of standard cells (AF488^−^PE^−^PI^+^APC^+^), the absolute number of viable Vγ9 T cells (AF488^+^PE^+^PI^−^) in a given microculture well was determined as described previously [12,31]. Expansion ratios were calculated by dividing the number of Vγ9 T cells determined by SCDA after 7 days of culture by the number of Vγ9 T cells calculated to be present within the starting PBMC responder cell population.

### 2.4. Flow Cytometry

The following mAb were obtained from BD Biosciences (Heidelberg, Germany): anti-CD3-PE/APC/BV605 (clone SK7), anti-CD14-FITC/APC (clone MoP9), anti-PD-L1-BV421 (clone MIH1), and anti-IFN-γ-PE (clone 4S.B3). Anti-Vδ2-FITC (clone IMMU389) was obtained from Beckman Coulter (Krefeld, Germany), anti-CD277-PE (clone BT3.1) from BioLegend (San Diego, CA, USA). For cell surface staining, 3 × 10^5^ cells were washed, stained for 20 min on ice with mAb, washed twice, and resuspended in 1% paraformaldehyde. For intracellular staining, cells were permeabilized in Cytofix/Cytoperm buffer (BD Biosciences) before staining with fluorochrome-conjugated mAb. All analyses were measured on a FACS Calibur or Fortessa cytometer (BD Biosciences), using the software Cell–Quest™ Pro and DIVA (Data-Interpolating Variational Analysis) for acquisition respectively, and FlowJo™ v10.6.1 software for data analysis.

### 2.5. Cell Death Analysis

Cell death of purified monocytes cultured overnight in the absence or presence of TLR ligands was measured by flow cytometry following combined annexin V-FITC/PI staining. In addition, cell cultures were subjected to microscopic inspection and photographs were taken with an Axiowert 10 microscope (Leitz, Wetzlar, Germany) equipped with an Axiocam 105 camera device and ZEN 2 core v2.5 software. For this purpose, 1.5 × 10^5^ purified monocytes per well were cultured in 96-well flat-bottom microtiter plates.

### 2.6. Measurement of Cytokines in Cell Culture Supernatants

IFN-γ, IL-1β, and TNF-α were quantified in cell culture supernatants by ELISA with the respective DuoSet ELISA Kits from R&D Systems (Wiesbaden, Germany) following the manufacturer’s instructions.

### 2.7. Statistical Analysis

Statistical comparisons were made between two groups using one-or two- way ANOVA analysis and Dunnett’s multiple comparison test against respective internal controls. All analyses were done with the Graphpad Prism 8 software. Levels of significance were set as * *p* < 0.05, ** *p* < 0.01, *** *p* < 0.001 and **** *p* < 0.0001.

## 3. Results

We studied the effects of TLR7 and TLR8 ligands on the in vitro activation of human Vγ9Vδ2 γδ T cells using two different read-out systems, i.e., the rapid induction of IFN-γ production and the cellular expansion in response to stimulation with ZOL and pAg. Since ZOL- and pAg-reactive γδ T cells always co-express Vγ9 and Vδ2, these cells are hereafter referred to as Vγ9 or Vδ2, depending on the antibodies used for staining.

### 3.1. TLR8 but not TLR7 Ligands Stimulate IFN-γ and Synergize with Phosphoantigen (E)-4-Hydroxy-3-Methyl-but-2-Enyl Pyrophosphate (HMBPP)-Induced IFN-γ Production in Vδ2 T Cells

**Figure 1:**
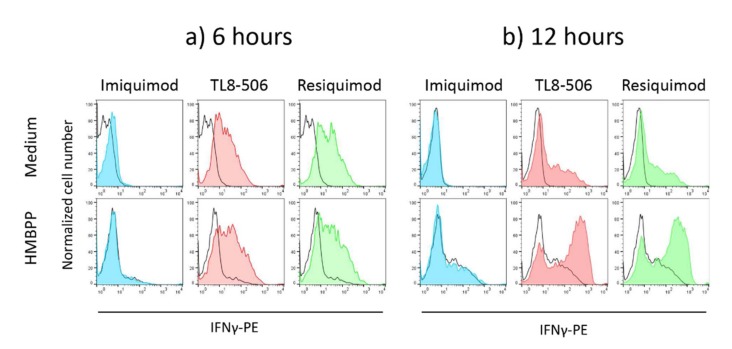
TLR8 and TLR7/8, but not TLR7 ligands, induce and co-stimulate IFN-γ production in Vδ2 T cells. Peripheral blood mononuclear cells (PBMC) from healthy donors were stimulated for 6 (**a**) or 12 h (**b**) with 1 μg/mL Imiquimod (TLR7 ligand), 0.1 μg/mL TL8-506 (TLR8 ligand), or 1 μg/mL Resiquimod (TLR7/8 ligand) in the absence (upper panel) or presence (lower panel) of 10 nM HMBPP. Monensin was present during the last 4 h of stimulation. Thereafter, cells were surface stained with anti-CD3 and anti-Vδ2 mAb, and permeabilized before staining with anti-IFN-γ mAb. A gate was set on Vδ2-positive cells. Black lines indicate the controls (medium only in upper row, medium with HMBPP in lower row). Results are representative of four independent experiments.

PBMC from healthy donors containing on average 2–4% Vδ2 T cells were stimulated for 6 to 24 h with 1 μg/mL TLR7 ligand Imiquimod, 0.1 μg/mL TLR8 ligand TL8-506, or 1 μg/mL TLR7/8 ligand Resiquimod in the absence of presence of 10 nM HMBPP. Cultures set up in medium only served as a control. Intracellular expression of interferon-γ (IFN-γ) in Vδ2 T cells was determined after 6 and 12 h (following 4 h incubation with monensin), and IFN-γ present in cell culture supernatants was measured after 24 h by ELISA. In the absence of TLR ligands or HMBPP, no intracellular IFN- could be detected (Figure 1a, upper row, black line). However, as shown in a representative experiment depicted in Figure 1a, TL8-506 and Resiquimod, but not Imiquimod, induced IFN-γ expression in Vδ2 T cells within PBMC in the absence of pAg HMBPP (Figure 1a, upper row) already after 6 h. Moreover, both TLR8 and TLR7/8, but not TLR7 agonists, also synergized with the HMBPP-triggered IFN-γ expression in Vδ2 T cells after 12 h (Figure 1b, lower row). At the early time point of 6 h, there was only little induction of IFN-γ by HMBPP in the absence of TLR ligands (black line in Figure 1a, lower row; compare with black line in Figure 1a, upper row [medium only]). On the other hand, the intracellular expression of IFN-γ induced by TLR ligands alone was less after 12 h compared to 6 h (Figure 1b, upper row). The intracellular IFN-γ expression as revealed by flow cytometry corresponded to the levels of secreted IFN-γ detected in cell culture supernatants by ELISA. As shown in Figure S1a, both TL8-506 and Resiquimod (but not Imiquimod) strongly enhanced the levels of IFN-γ in supernatants of PBMC activated with the Vδ2 T-cell-specific pAg HMBPP. Additional experiments with purified γδ T cells co-cultured with purified monocytes and activated with HMBPP proved that IFN-γ was indeed produced and secreted by γδ T cells (Figure S1b).

### 3.2. TLR8 Ligands Inhibit Cellular Expansion of Vδ2 T Cells in Response to Zoledronic Acid (ZOL) and HMBPP

The above results showed that TLR8 ligands can potently co-stimulate the in vitro activation of Vδ2 T cells as measured by the early induction of IFN-γ production. Proliferative expansion is another consequence of T-cell activation. We next investigated the modulation of Vδ2 T-cell expansion in response to selective activation stimuli. To this end, we stimulated PBMC (containing on average 2 to 4% γδ T cells) or purified γδ T cells together with purified monocytes with ZOL or pAg HMBPP in the presence of IL-2, and additional presence of absence of TLR ligands. We measured the cellular expansion after 6 to 7 days by a flow cytometry method and determined the absolute number of viable Vδ2 T cells per microculture well. There was substantial variability among healthy blood donors in their capacity to expand γδ T cells in vitro. To normalize the results obtained with different blood donors, we calculated the expansion index based on the known proportion of Vδ2 T cells in the starting responder cell populations. To this end, the number of viable γδ T cells per microwell after the culture period was divided by the calculated number of γδ T cell at the initiation of cell culture.

**Figure 2:**
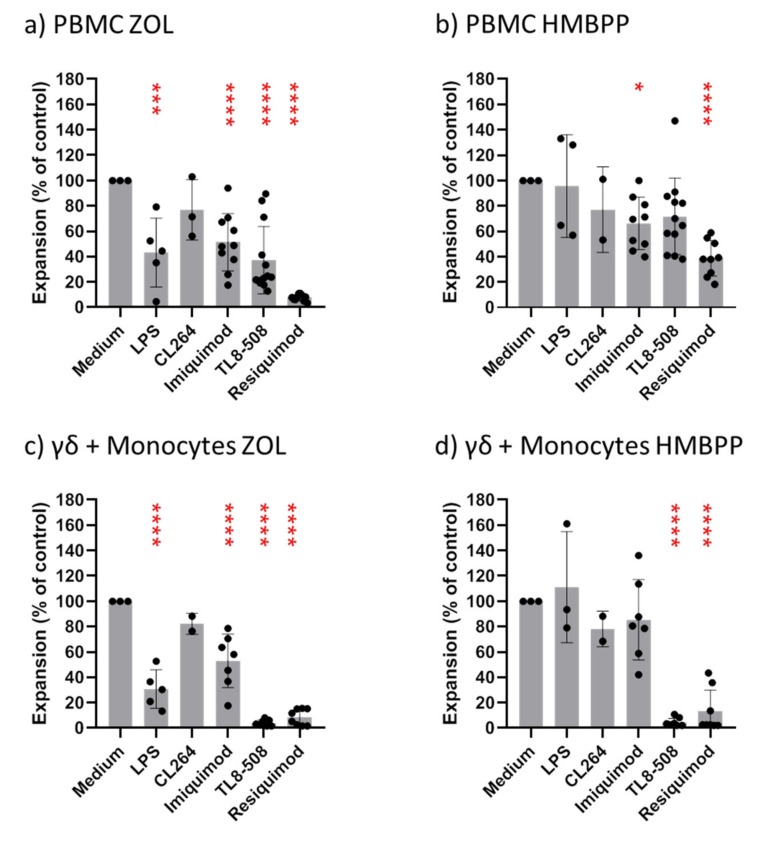
Modulation of Vγ9 T-cell expansion by toll-like receptor (TLR) ligands. 1.5 × 10^5^ PBMC (**a,b**) or 2 × 10^4^ purified γδ T cells plus 5 × 10^4^ purified monocytes (**c,d**) obtained from healthy donors were cultured per microwell in 96-well round-bottom plates and were stimulated with 2.5 μM zoledronic acid (ZOL) (**a,c**) or 10 nM HMBPP (**b,d**) in the presence of 50 IU/mL IL-2. Cultures were supplemented with TLR ligands as indicated (0.1 μg/mL LPS, 1 μg/mL CL264 or Imiquimod, 0.1 μg/mL TL8-506, or 1 μg/mL Resiquimod). All cultures were set up in duplicates. After 6–7 days (PBMC) or 8 days (γδ T cells plus monocytes), absolute numbers of viable Vγ9 T cells were measured by standard cell dilution assay (SCDA). Expansion index was calculated by dividing the measured number of Vγ9 T cells after culture by the calculated number of Vγ9 T cells at the start of the cell culture. The expansion index of Vγ9 T cells cultured in the absence of TLR ligands (medium only) was set to 100%. (**a,b**) Mean + SE of 5 (LPS), 3 (CL264), 10 (Imiquimod), 13 (TL8-506), and 10 (Resiquimod) experiments with different blood donors. (**c,d**) Mean + SE of 5 (LPS), 2 (CL264), 6 (Imiquimod), 8 (TL8-506) and 6 (Resiquimod) experiments with different blood donors. * *p* < 0.05, *** *p* < 0.001, **** *p* < 0.0001.

When PBMC were stimulated with ZOL and IL-2, Vδ2 T cells expanded within 6–7 days between 25- and 210-fold (mean 56.3; *n* = 13), and stimulation with HMBPP and IL-2 induced 13- to 159-fold expansion (mean 49.1; *n* = 12) of Vδ2 T cells. The addition of predetermined optimal concentrations of ligands for TLR4 (ultrapure LPS; 0.1 μg/mL), TLR7 (CL264, Imiquimod; both at 1 μg/mL), TLR8 (TL8-506; 0.1 μg/mL), or TLR7/8 (Resiquimod; 1 μg/mL) at the initiation of cell cultures all reduced the expansion of Vδ2 T cells within PBMC stimulated with ZOL (Figure 2a). Inhibition was most pronounced with Resiquimod (7.5% of control), but substantial inhibition also occurred with TL8-506 (37% of control), TLR7 ligands (Imiquimod: 55% of control; CL264: 77% of control) and LPS (53% of control). Interestingly, the proliferative expansion of Vδ2 T cells within PBMC in response to HMBPP plus IL-2 was less susceptible to inhibition by TLR ligands (Figure 2b). Again, however, Resiquimod was the most potent inhibitor (39% of control), whereas the other ligands resulted in 66% (Imiquimod) to 96% (LPS) of control proliferation (Figure 2b).

The selective inhibition of Vγ9 T-cell expansion by TLR8 and TLR7/8 ligands (TL8-506, Resiquimod) was much more evident when purified γδ T cells together with monocytes rather than PBMC were stimulated with ZOL (Figure 2c) and particularly with HMBPP (Figure 2d). In response to ZOL stimulation, TL8-506 suppressed the Vγ9 T cell expansion to 4% of control, and Resiquimod to 9% of control, whereas Vγ9 T-cell expansion in the presence of LPS, CL264, or Imiquimod was 31%, 82%, and 59% of control, respectively. The results were even more clear-cut when γδ T cells plus monocytes were stimulated with HMBPP rather than ZOL (Figure 2d). Both TL8-506 and Resiquimod strongly inhibited Vγ9 T-cell expansion (5% of control with both ligands), whereas LPS did not inhibit at all, and Imiquimod had only a minor effect (87% of control). The suppression of Vδ2 T-cell growth by TL8-506 and Resiquimod was clearly monocyte-dependent. When high numbers of purified γδ T cells (10^5^ per well) were stimulated with HMBPP and IL-2 in the absence of monocytes, the addition of TL8-506 or Resiquimod did not have any inhibitory effect (not shown).

**Figure 3:**
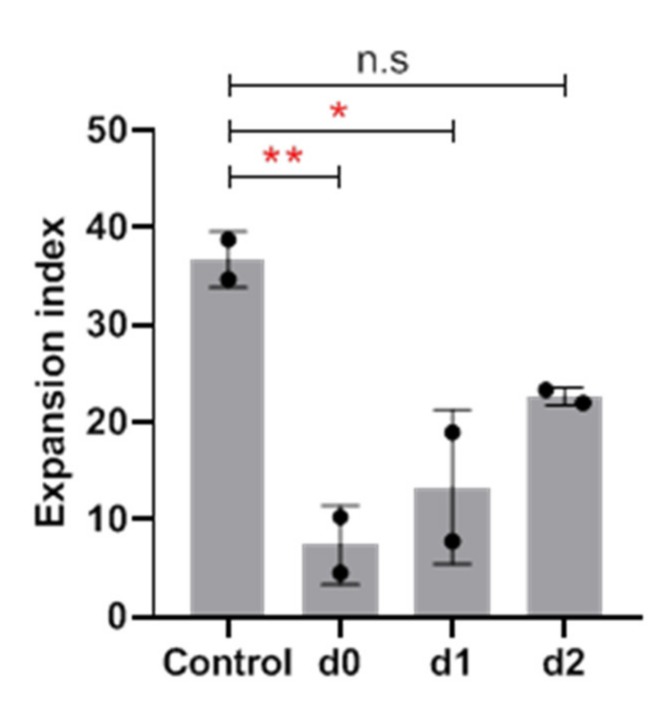
Time dependency of the inhibitory effect of Resiquimod on Vγ9 T-cell expansion. 1.5 × 10^5^ PBMC were stimulated in duplicates with 2.5 μM ZOL in the presence of 50 IU/mL IL-2 (control). 1 μg/mL Resiquimod was added at the initiation of cell cultures (d0), or 1 or 2 days later (d1, d2). Control cultures were set up without Resiquimod. After 6 days, absolute numbers of viable Vγ9 T cells were measured by SCDA. Expansion index was calculated by dividing the measured number of Vγ9 T cells after culture by the calculated number of Vγ9 T cells at the start of the cell culture. Results are the mean + SE of two separate experiments. Statistical significance was assessed by Dunnett’s multiple comparison test. * *p* < 0.5, ** *p* < 0.01.

Moreover, the inhibitory TLR ligands had to be added at the initiation of cell cultures to exert the suppressive effect. As shown in Figure 3, the delayed addition of Resiquimod after one and particularly after two days abrogated the inhibitory effect. Taken together, these results clearly demonstrate that the proliferative expansion of freshly isolated γδ T cells is potently suppressed by TLR8 ligands which need to be added at the set-up of cell cultures for maximal inhibition.

### 3.3. TLR8 Ligands Abrogate the Monocyte-Dependent Proliferation of Short-Term Expanded Vδ2 T-Cell Lines in Response to Phosphoantigen Bromohydrin Pyrophosphate (BrHPP)

In view of the monocyte-dependent inhibitory effect of TLR8 and TLR7/8 ligands on the ZOL- and HMBPP- stimulated proliferation of freshly isolated γδ T cells, we next investigated the effect of those TLR ligands on the proliferative expansion of short-term activated and expanded Vγ9Vδ2 γδ T-cell lines re-stimulated with the pAg BrHPP in the absence of presence of purified monocytes. Again, cellular expansion was quantified by SCDA. γδ T-cell lines originating from stimulation of PBMC with ZOL and repeated addition of IL-2 routinely contained >95% Vγ9Vδ2 T cells after 14 days [30]. Such Vδ2 T-cell lines were washed, re-seeded at 5 × 10^4^ cells per well in the presence of 50 IU/mL IL-2 and presence of absence of 300 nM pAg BrHPP and/or 5 × 10^4^ freshly isolated monocytes. TLR8 ligands (TL8-506, 0.1 μg/mL; Motolimod, 0.5 μg/mL) or TLR7/8 ligand Resiquimod (1 μg/mL) where added as indicated, and absolute numbers of viable cells were determined by SCDA after 3 and 6 or 7 days.

**Figure 4:**
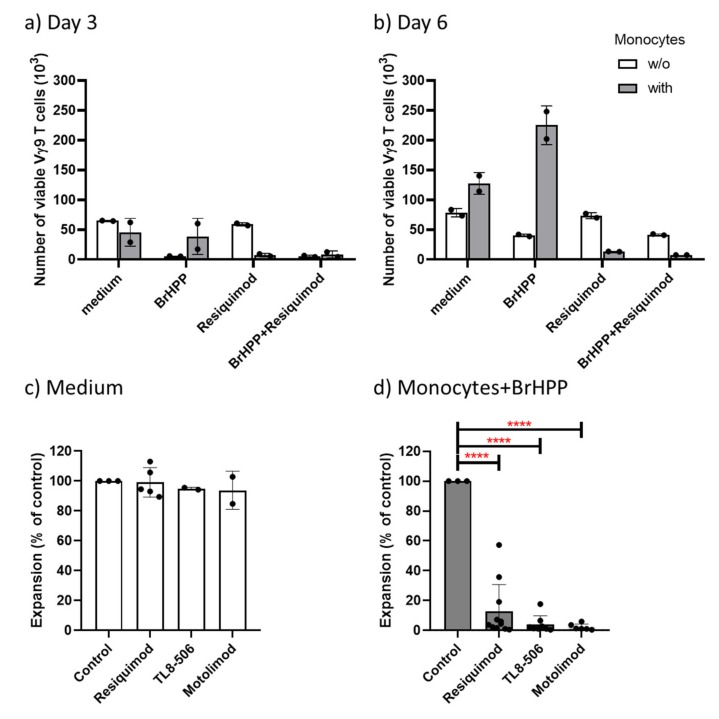
TLR8 ligands suppress the monocyte-dependent re-stimulation of Vγ9Vδ2 T-cell lines with bromohydrin pyrophosphate (BrHPP). 5 × 10^4^ γδ T-cell lines (>95% CD3^+^Vδ2^+^) cells were cultured in 50 IU/mL L-2 with (grey columns) or without (open columns) 5 × 10^4^ purified monocytes in the absence of presence of 300 nM BrHPP and Resiquimod (1 μg/mL), TL8-506 (0.1 μg/mL) or Motolimod (0.5 μg/mL) as indicated. The absolute number of viable Vγ9 T-cells was determined after 3 or 6/7 days by SCDA. All cultures were set up in duplicates. (**a**) and (**b**) Results (absolute cell numbers per well; mean of duplicates) of a representative experiment are shown for day 3 (**a**), and for day 6 (**b**). (**c**) Results with additional Vδ2 T-cell lines cultured without monocytes and with or without TLR ligands; (**d**) Results with the same Vδ2 T-cell lines in the additional presence of monocytes and BrHPP. In (**c**) and (d), cell numbers of the respective control (c: medium only; d: monocytes plus BrHPP) were set to 100%, and results are displayed as % of controls. c: Resiquimod (*n* = 5), TL8-506 (*n* = 2), Motolimod (*n* = 2). d: Resiquimod (*n* = 9), TL8-506 (*n* = 7), Motolimod (*n* = 6). **** *p* < 0.0001.

Results of a representative experiment showing the absolute numbers of viable Vγ9 T cells after culture are displayed in Figure 4a and b. In the absence of monocytes, BrHPP efficiently induced activation-induced cell death as reflected by the strongly reduced Vγ9 T-cell number on day 3 (Figure 4a) and day 6 (Figure 4b) when compared to medium controls (open columns). The addition of Resiquimod alone did not modulate Vγ9 T-cell proliferation, neither on day 3 (Figure 4a) nor on day 6 (Figure 4b), in line with lack of a direct effect on γδ T cells. When monocytes were present (grey columns), BrHPP-induced activation-induced cell death on day 3 was strongly prevented (as evidenced by the larger number of viable Vγ9 T cells; Figure 4a), and in fact the presence of monocytes enabled potent expansion of Vγ9 T cells re-stimulated with BrHPP, as measured after 6 days (Figure 4b). This rescuing effect of monocytes was almost completely abolished in the presence of Resiquimod, both on day 3 (Figure 4a) as well as on day 6 (Figure 4b). Such experiments were additionally also performed with TL8-506 and the TLR8 ligand Motolimod, and a summary of 6 to 9 experiments performed with γδ T-cell lines established from different donors is presented in Figure 4c, d. The presence of Resiquimod, TL8-506 or Motolimod did not significantly affect the proliferation of Vγ9 T-cell lines in the absence of monocytes and BrHPP (99%, 95%, and 94% of controls, respectively; Figure 4c). In contrast, all three ligands strongly suppressed the proliferative expansion of Vγ9 T-cell lines re-stimulated with BrHPP in the presence of monocytes (13%, 4%, and 2% of control in the presence of Resiquimod, TL8-506, and Motolimod, respectively; Figure 4d).

### 3.4. TLR8 Ligands Potently Stimulate IL-1β and TNF-α Secretion in Monocytes but Simultaneously Induce Monocyte Death

As shown above, TLR8 ligands co-stimulate IFN-γ induction in a monocyte-dependent manner in resting γδ T cells but simultaneously suppress their cellular expansion. Therefore, we next investigated the direct effect of the various TLR ligands on purified monocytes at the level of IL-1β/TNF-α secretion, modulation of certain cell surface molecules, and cell death induction.

**Figure 5:**
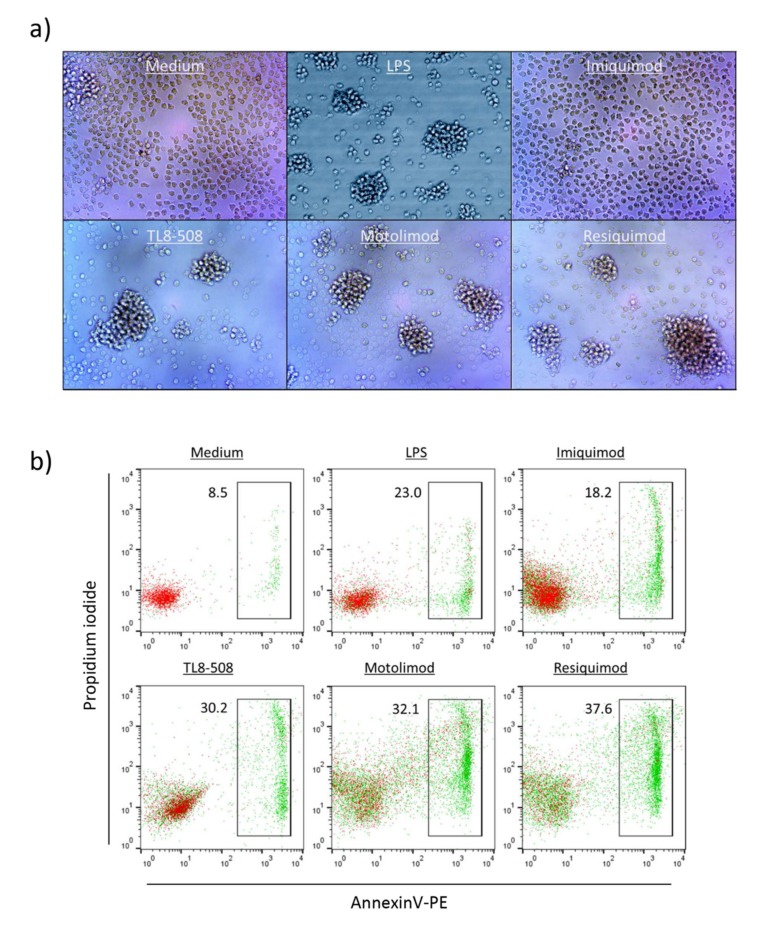
TLR8 ligands induce cell death in purified monocytes. 1.5 × 10^4^ purified monocytes per well were stimulated in flat-bottom 96-well plates for 24 h with LPS (0.1 μg/mL), Imiquimod (1 μg/mL), Resiquimod (1 μg/mL), TL8-506 (0.1 μg/mL) or Motolimod (0.5 μg/mL) as indicated. Microscopic inspection (**a**) was done after 24 h at 100x magnification. Annexin V/PI staining was performed in parallel and analyzed by flow cytometry (**b**). Results are representative of four independent experiments.

**Figure 6:**
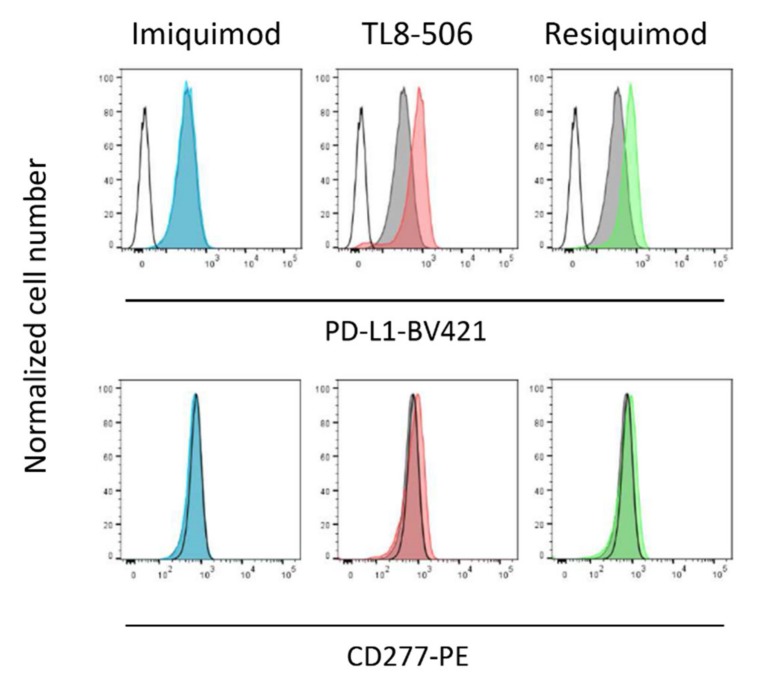
TLR8 and TLR7/8 but not TLR7 ligands up-regulate PD-L1 expression on monocytes. Purified monocytes were cultured for 12 h in medium supplemented or not with 1 μg/mL Imiquimod, 0.1 μg/mL TL8-506, or 2 μg/mL Resiquimod. Thereafter, cells were stained with anti-PD-L1-BV421 (upper row) and anti-CD277-PE (lower row) as indicated. Open black histogram: staining of freshly isolated monocytes. Filled grey histograms: medium control after 12 h. Colored histograms: Staining after incubation with respective ligands. Results of one out of two experiment are shown.

As shown in Figure S2, both Motolimod as well as Resiquimod and an additional TLR8 RNA ligand (ssRNA40) (but not the negative control ssRNA41) strongly induced IL-1β (Figure S2a) as well as TNF-α secretion (Figure S2b) in purified monocytes. At the same time, however, TLR8 (but not TLR7) ligands induced substantial monocyte cell death after 24 h as determined by microscopical inspection (Figure 5a) and combined Annexin V/PI staining (Figure 5b). While the microscopic pictures clearly showed massive cell death, they also demonstrated intensive cluster formation of surviving monocytes induced by the active ligands, but not by Imiquimod. Importantly, we also observed modulatory effects of the active ligands TL8-506 and Resiquimod (but not of Imiquimod) on the cell surface expression of PD-L1 on surviving monocytes. Freshly isolated monocytes do not express PD-L1 but upregulate PD-L1 upon overnight culture in serum-containing medium [32,33]. As illustrated in Figure 6, negatively isolated monocytes expressed PD-L1 after overnight culture when compared to freshly isolated cells (upper row: grey histograms versus open histogram). Both TL8-506 and Resiquimod (but not Imiquimod) induced some up-regulation of PD-L1 after overnight incubation (Figure 6, upper row), but did not modulate cell surface expression of BTN3A1/CD277 (Figure 6, lower row).

## 4. Discussion

TLRs are a major class of pattern recognition receptors which are predominantly expressed in innate immune cells; however, at least some TLRs are also functionally expressed in adaptive immune cells like B cells and T cells. As a consequence, certain TLR ligands can modulate T-cell activation either directly or indirectly via accessory cells [34,35]. Human γδ T cells also express some TLRs, like for instance TLR3, and corresponding ligands can directly modify γδ T-cell activation [26,36]. In addition, indirect effects of ligands for some TLR, like TLR2 or TLR4 on γδ T-cell activation, mediated via accessory cells, have been reported [37]. In this study we focused on TLR7 and TLR8, as ligands for these two receptors are in clinical use or development for cancer therapy. The TLR7 ligand Imiquimod has been approved by the US Food and Drug Administration (FDA) for the treatment of actinic keratosis, external genital/perianal warts (condylomata acuminata), and superficial basal cell carcinoma, and is in clinical studies for other cancers, as is the TLR8 agonist Motolimod [38]. The TLR7/8 agonist Resiquimod is in clinical studies for treatment of cutaneous T-cell lymphoma [39]. Additional experimental studies suggest that the local application of TLR7/8 agonists in combination with other immunotherapies might have beneficial effects [23].

Modulation of T-cell activation with Resiquimod has been previously studied, but not with respect to human γδ T cells. Richard-Pargmann and co-workers reported a co-stimulatory effect of Resiquimod and TLR8-specific oligonucleotides on the activation of purified human T cells by anti-CD3 mAb which, however, was abrogated in the presence of monocytes [24]. In line, a suppressive activity of Resiquimod-treated monocytes on human CD4 T-cell activation was also reported by Giesbrecht et al. [25]. In both studies, Resiquimod did not appear to induce cell death of monocytes. Rather, it was suggested that Resiquimod reprogrammed human monocytes in an IL-1β dependent manner into suppressive cells that inhibited CD4 T-cell activation via up-regulation of PD-L1, adenosine, and indoleamine 2,3-dioxygenase activity [25].

In our experiments we observed that the TLR7/8 agonist Resiquimod and the more specific TLR8 ligands TL8-506 and Motolimod, but not TLR7 ligand Imiquimod, stimulated early IFN-γ expression in Vδ2 T cells when PBMC were activated by TLR ligands in the absence of additional γδ T-cell stimuli. Moreover, TLR8 ligands potently co-stimulated the pAg-induced IFN-γ production in the Vδ2 T cells. This effect was monocyte dependent as shown by cell fractionation experiments. On the other hand, the very same TLR8 ligands potently suppressed the in vitro expansion of Vδ2 T cells in two different experimental settings, i.e., the monocyte-dependent expansion of freshly isolated γδ T cells in response to ZOL or HMBPP, as well as the re-stimulation of short-term expanded Vδ2 T-cell lines with pAg BrHPP, again in the presence of monocytes. Importantly, the TLR ligands per se did not affect the viability of either freshly isolated γδ T cells or short-term expanded Vδ2 T-cell lines. Cell separation and reconstitution experiments clearly proved that the inhibitory effect required the presence of monocytes. Further studies of the direct effects of TLR8 ligands on isolated monocytes revealed potent induction of IL-1β and TNF-α production, but at the same time also induction of monocyte cell death. These results indicate that the TLR8 ligand-activated monocytes provide potent co-stimulation for IFN-γ production by freshly isolated γδ T cells. The detection of large amounts of IL-1β in the supernatants of TLR8-activated monocytes suggests that IL-1β might be involved in mediating this co-stimulation—a notion which is supported by the studies of Giesbrecht et al. [25], but which requires further investigation.

In contrast to previous studies which did not observe massive cell death of human monocytes exposed to Resiquimod [24,25], we noticed that those ligands which co-stimulated IFN-γ induction and suppressed γδ T-cell expansion, i.e., Resiquimod, Motolimod, TL8-506, always induced significant cell death in monocytes as revealed by microscopic inspection and combined Annexin V/PI staining. Activation of the inflammasome in monocytes activates NLRP3 and Caspase-1 leading to IL-1β secretion and inflammatory cell death termed pyroptosis [17]. This includes a priming step where TLR4 ligand LPS (or TLR2 ligands) up-regulate pro-IL-1β, and stimulation with e.g., the bacterial toxin nigericin activates NLRP3 leading to cytokine secretion. However, human monocytes can also undergo an alternative NLRP3 activation associated with pyroptosis-independent IL-1β secretion [40]. TLR8 ligands can stimulate NLRP3-dependent IL-1β as well as NLRP3-independent TNF-α secretion in human monocytes [41]. Since imidazoquinoline compounds like Resiquimod also activate the NRLP3 [41], we assume that TLR8-stimulated monocytes undergo pyroptosis. Why do dying monocytes so potently inhibit γδ T-cell expansion? With regard to the activation of freshly isolated γδ T cells (or γδ T cells within PBMC) with ZOL, one could consider that TLR8 activation might interfere with the production of the γδ T cell-stimulating endogenous IPP [2]; this, however, should not be relevant for the stimulation with the pAg HMBPP. Moreover, in the presence of Resiquimod, monocytes also abrogated the expansion of short-term expanded Vδ2 T-cell lines in response to re-stimulation with the pAg BrHPP, which again is independent of ZOL-mediated IPP production. This proliferation of activated Vδ2 T cells re-stimulated by BrHPP is dependent on viable monocytes as accessory cells; in the absence of monocytes, BrHPP and related TCR stimuli induce activation-induced cell death of γδ T cells [42,43,44]. In the presence of monocytes, activation-induced cell death of T cells is drastically reduced, and surviving T cells are rescued for vigorous proliferative response to antigenic restimulation [45]. Therefore, a simplistic explanation for the inhibition of γδ T-cell expansion by TLR8 ligands is that monocytes undergo pyroptosis and, as a consequence, there are not enough viable monocytes around to serve as accessory cells for the proliferative activation of resting or activated/already expanded Vδ2 T cells. Currently, however, we cannot exclude that the dying or the surviving monocytes also somehow actively suppress γδ T-cell activation. We noticed that despite massive cell death, surviving monocytes formed cell clusters in response to TLR8 ligands (Figure 5a). Moreover, we observed that TL8-506 and Resiquimod (but not Imiquimod) slightly up-regulated PD-L1 expression on surviving monocytes but did not modulate CD277/BTN3A1 expression. PD-L1 on monocytes is known to suppress T-cell activation [32,33,46], and Resiquimod-induced upregulation of PD-L1 on monocytes was one of several inhibitory mechanisms identified by Giesbrecht et al. [22]. However, we believe that additional mechanisms are at play in the inhibition of γδ T-cell proliferation, since in contrast to our study, Giesbrecht et al. [22] did not observe induction of monocyte cell death upon exposure to Resiquimod.

## 5. Conclusions

Our results add to the complexity of the modulation of human γδ T-cell activation by TLR ligands. Resiquimod, Motolimod, and perhaps other TLR8 ligands inhibit γδ T-cell expansion in the presence of monocytes and are thus to be excluded from protocols aiming at large scale expansion of γδ T cells in vitro. On the other hand, the very same TLR8 ligands are potent (again monocyte-dependent) co-stimuli for activation (e.g., IFN-γ production) of γδ T cells. This may be useful for boosting anti-tumor activity in γδ T cells when TLR8 ligand adjuvants are applied locally into the tumor microenvironment, as proposed in clinical studies [47].

## Supplementary Materials

The following are available online at https://www.mdpi.com/2073-4409/9/3/713/s1, Figure S1: Secretion of IFNγ in cell culture supernatants of PBMC and γδ T-cell/monocyte co-cultures. Figure S2: Secretion of IL-1β and TNF-α in cell culture supernatants of purified monocytes.
