# Correction: Serrano, R.; Wesch, D.; Kabelitz, D. Activation of Human γδ T Cells: Modulation by Toll-Like Receptor 8 Ligands and Role of Monocytes. *Cells* 2020, *9*, 713

**DOI:** 10.3390/cells9091977

**Published:** 2020-08-27

**Authors:** Ruben Serrano, Daniela Wesch, Dieter Kabelitz

**Affiliations:** Institute of Immunology, University Hospital Schleswig-Holstein Campus Kiel, D-24105 Kiel, Germany; ruben.serrano@uksh.de (R.S.); daniela.wesch@uksh.de (D.W.)

The authors wish to make the following changes to their paper [1].

TL8-506 was wrongly labeled as TL8-508 in Figures 2 and 5 of the original and should be replaced with:

**Figure 2**: 
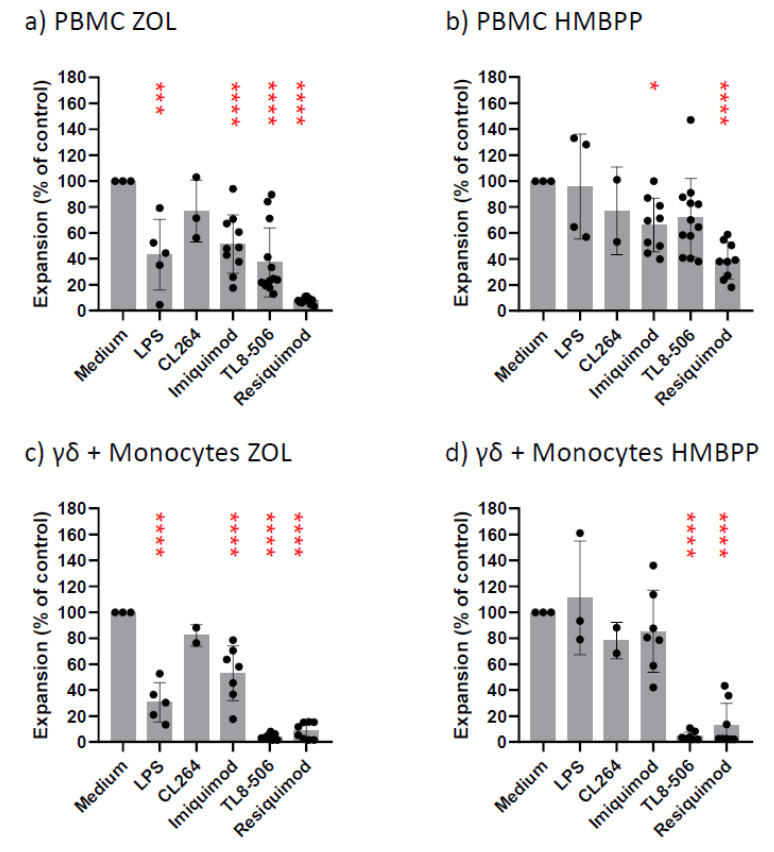


**Figure 5**: 
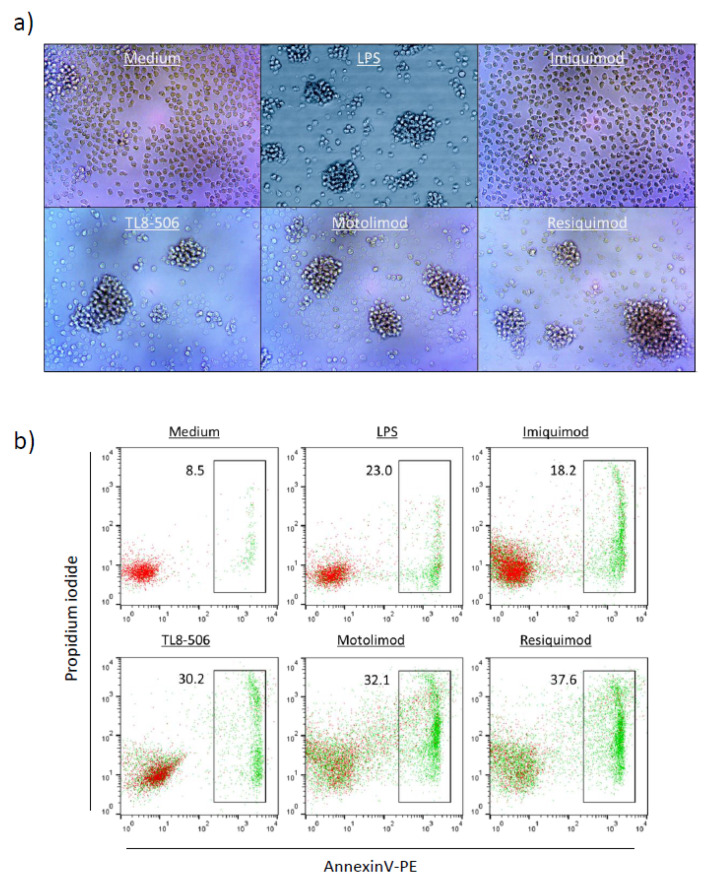


The authors would like to apologize for any inconvenience caused to the readers by these changes. The changes do not affect the scientific results. The manuscript will be updated and the original will remain online on the article webpage, with a reference to this correction.
