# Supplementary material for: Activation of Human γδ T Cells: Modulation by Toll-Like Receptor 8 Ligands and Role of Monocytes
Source: Cells. 2020 Mar 13;9(3):713. doi: 10.3390/cells9030713 (PMC7140608; doi:10.3390/cells9030713)
Supplement: Supplementary file 1 [file cells-09-00713-s001.pdf]

Supplemental Figure S1

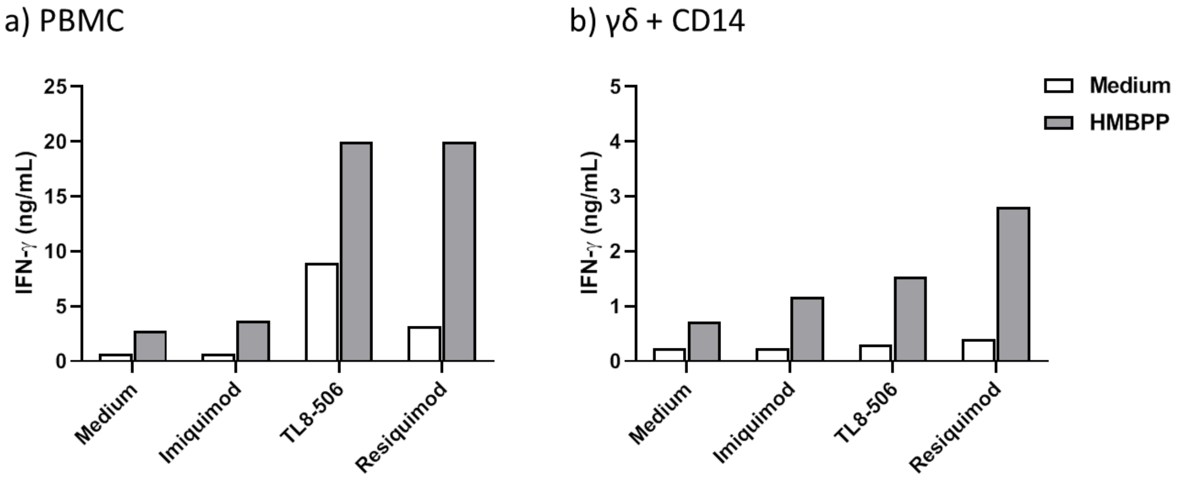

Supplemental Figure S1: Secretion of IFNγ in cell culture supernatants of PBMC and γδ T-cell/monocyte co-cultures. 1.5x10^5 PBMC (a), or 2x10^4 purified γδ T cells plus 5x10^4 purified monocytes (b) from a healthy donor were activated with TLR ligands Imiquimod (1μg/mL), TL8-506 (0.1μg/mL) or Resiquimod (1μg/mL) as indicated. HMBPP was added at 10nM to PBMC (a) and to γδ T cell/monocyte co-cultures (b) where indicated. After 24hr, cell culture supernatants were collected and stored at -20°C until quantification of IFN-γ by ELISA. Mean of duplicate determinations, results of one representative experiment.

Supplemental Figure S2

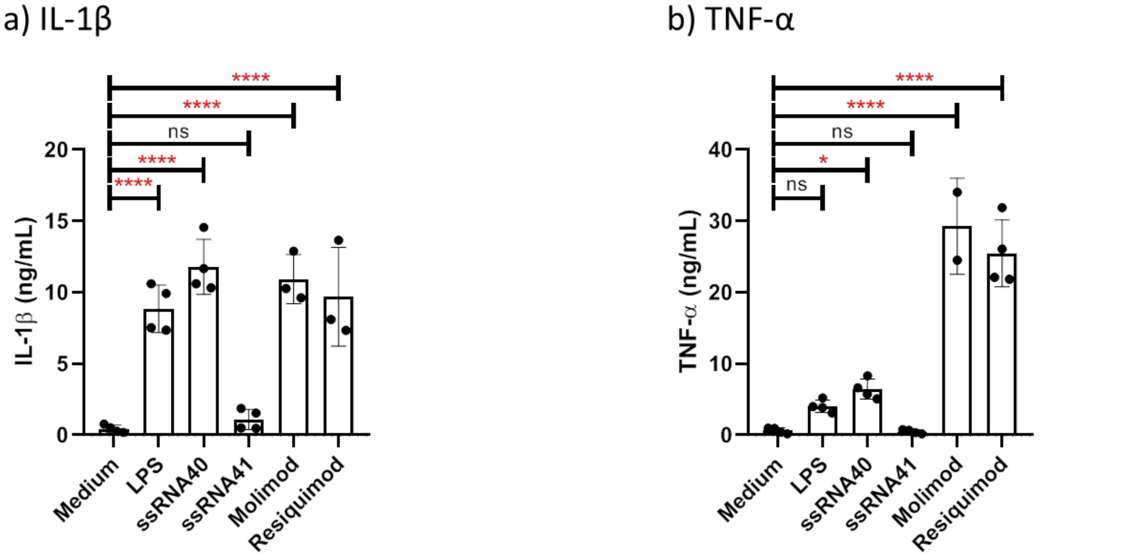

Supplemental Figure S2: **Secretion of IL-1β and TNF-α in cell culture supernatants of purified monocytes.** Monocytes were purified from PBMC obtained from healthy blood donors by negative magnetic isolation. 10^5 monocytes/well were cultured in 96-well round-bottom microtiter plates in RPMI with 10% heat-inactivated low endotoxin FBS and in the presence or absence of 0.1 µg/mL LPS, 1 µg/mL ssRNA40/41, 0.5 µg/mL Motolimod, or 1 µg/mL Resiquimod. After 24hr, supernatants were collected and stored at -20°C until quantification of IL-1-β (a) and TNF-α (b) by ELISA. Mean ± SE of two to four individual experiments. n.s., not significant, * $p<0.05$, **** $p<0.0001$
